# SLIPTA e-Tool improves laboratory audit process in Vietnam and Cambodia

**DOI:** 10.4102/ajlm.v3i2.219

**Published:** 2014-11-03

**Authors:** Thuong T. Nguyen, Barbara McKinney, Antoine Pierson, Khue N. Luong, Quynh T. Hoang, Sandeep Meharwal, Humberto M. Carvalho, Cuong Q. Nguyen, Kim T. Nguyen, Kyle B. Bond

**Affiliations:** 1 National Institute of Hygiene and Epidemiology (NIHE), Vietnam; 2 Mayo Clinic, United States; 3 Integrated Quality Laboratory Services (IQLS), France; 4 Vietnam Administration for Medical Services (VAMS), Vietnam; 5 US Centers for Disease Control and Prevention (CDC), Vietnam; 6 United States Agency for International Development (USAID) Deliver Project, Indonesia; 7 Substance Abuse and Mental Health Services Administration (SAMHSA), United States; 8 FHI360, Vietnam; 9 French Department, Hanoi University of Science and Technology, Hanoi, Vietnam

## Abstract

**Background:**

The Stepwise Laboratory Quality Improvement Process Towards Accreditation (SLIPTA) checklist is used worldwide to drive quality improvement in laboratories in developing countries and to assess the effectiveness of interventions such as the Strengthening Laboratory Management Toward Accreditation (SLMTA) programme. However, the paper-based format of the checklist makes administration cumbersome and limits timely analysis and communication of results.

**Development of e-Tool:**

In early 2012, the SLMTA team in Vietnam developed an electronic SLIPTA checklist tool. The e-Tool was pilot tested in Vietnam in mid-2012 and revised. It was used during SLMTA implementation in Vietnam and Cambodia in 2012 and 2013 and further revised based on auditors’ feedback about usability.

**Outcomes:**

The SLIPTA e-Tool enabled rapid turn-around of audit results, reduced workload and language barriers and facilitated analysis of national results. Benefits of the e-Tool will be magnified with in-country scale-up of laboratory quality improvement efforts and potential expansion to other countries.

## Introduction

The Stepwise Laboratory Quality Improvement Process Towards Accreditation (SLIPTA) checklist was established by the World Health Organization’s Regional Office for Africa (WHO AFRO) and partners to assess the level of a laboratory’s compliance with the International Organization for Standardisation (ISO) 15189 standard.^1^ This checklist is the standardised tool used to assess the effectiveness of the Strengthening Laboratory Management Toward Accreditation (SLMTA) training programme^2^ through audits at the start (baseline) and end (exit) of the programme. Audits are conducted using the paper-based SLIPTA checklist containing 111 major questions (and numerous, related sub-questions) divided into 12 sections; as of 2012 only the English version was available. To date, the checklist has been used in 617 laboratories implementing SLMTA in 47 countries.^3^ Data collected using the SLIPTA checklist reveal the current state of a laboratory’s quality management systems and identify gaps in the 12 Quality System Essential areas defined by the Clinical and Laboratory Standards Institute.^4^ Audit results are used to develop action plans and guide the selection of quality improvement activities for the SLMTA programme so as to help laboratories move toward national or international accreditation.

The SLMTA programme in Vietnam and Cambodia began in 2010 when representatives from each country participated in a two-week Training-of-Trainers workshop. After stakeholder engagement, governmental approval and, in the case of Vietnam, planning with international partners, baseline audits were conducted in seven laboratories in Cambodia in June of 2011 using the standard paper-based SLIPTA checklist. Auditors and implementing leadership from Vietnam noted that using the paper-based checklist created several challenges. Cumbersome audit and administrative processes meant that auditors were required to bring blank paper-based checklists and, in the case of exit or follow-up audits, previously-completed checklists to review prior recommendations and scores. Scores for all 111 questions had to be calculated and/or graphed manually for each report in each round of audits. Additionally, communication opportunities were lost; data analysis was inefficient and delayed; reports were not standardised; and there were difficulties in combining data for regional- or country-level management.

As a result of the observed difficulties, prior to performing the baseline audits in Vietnam, Ministry of Health (MOH) laboratorians supporting SLMTA in Vietnam set out to develop an electronic tool (e-Tool) for collection and use of audit data. This paper describes the development of the SLIPTA checklist e-Tool and discusses its benefits for SLMTA implementation.

## Research methods and design

### Development of the e-Tool

Development of the SLIPTA checklist e-Tool began with analysis of the current audit workflow. Next, experts from the MOH and US Centers for Disease Control and Prevention’s Vietnam Office (CDC-Vietnam) gathered background information about the structure, scoring strategy and other aspects of the SLIPTA checklist from auditors and other stakeholders. The Tuberculosis Electronic Laboratory Assessment Tool (TB-LAT), a Microsoft^®^ Excel-based e-Tool developed previously by Integrated Quality Laboratory Services and used globally for tuberculosis assessment,^5^ was modified by the SLMTA Management Team within the Vietnam hospital administration system so as to incorporate the SLIPTA checklist. The structure of the checklist was retained. During the development of the e-Tool, input was sought from the MOH and CDC-Vietnam laboratorians working as SLMTA trainers and auditors in Vietnam.

The Vietnam SLMTA team pilot tested the e-Tool in early 2012 in parallel with the paper-based checklist in a provincial public health laboratory. The e-Tool was then used for the baseline audits of a mix of 12 public health, hospital and HIV laboratories in Vietnam in May 2012. The tool was further enhanced in an iterative process of feedback and improvement. Exit audits of the first round of SLMTA-supported laboratories in both Cambodia (January 2013) and Vietnam (June to July 2013) provided additional opportunities to refine the tool and validate macro formulae.

## Results

The baseline audit in Cambodia using the paper-based SLIPTA checklist had several limitations. Audits required at least one-and-a-half days, including two hours to manually fill out the 44-page SLIPTA checklist and calculate site scores in order to create the reports. Although audit teams gave verbal reports at the individual laboratory summation conferences immediately following the audits, final written reports to the laboratories were compiled remotely after the audit and delivered by mentors at their next visit, sometimes delayed as long as two weeks. Because the existing paper-based checklist is in a PDF or Microsoft^®^ Word format, multi-site data were not easily manipulated, reducing the analytical value. Additionally, the country team scanned and saved all final paper documents, requiring significant administrative time and costs for electronic data storage.

The e-Tool^6^ addresses several issues related to ease of use during the audit process and solutions for data management (Table 1). Drop-down response selections enable automatic scoring, easily accounting for non-applicable questions. Additional features of the electronic format include: automatic linkage of information; pre-programmed calculations to compare baseline and exit audits; and pre-set data check limits so as to help improve scoring accuracy. All comments entered in each of the 12 sections are automatically pulled to the end of the report to complete the recommendation section. Summary pages visually highlight the accomplishments and remaining gaps to be addressed, utilising clear colour-coded graphs.

**TABLE 1:** Challenges of the paper-based SLIPTA checklist, and solutions provided by the SLIPTA e-Tool.

Area for improvement	Challenges associated with paper-based checklist	e-Tool solutions
Audit process	Scoring errors when results are calculated manually	Automatically calculates scores as data are entered, with built-in data limit checks
	Length of time required for recording and entering data	On-site data entry into e-Tool reduces time to perform audit and eliminates the need to transfer data from hard copies into electronic format
Communication	Delays in reporting of results; manual calculations add at least one additional day to the audit process	Results are calculated on-site and presented immediately after audit
	Hospital administration is often not available at the follow-up visit; delays reduce motivation for improvement	Results are available on-site in real time while hospital administrators and laboratorians are present and highly motivated to make further improvements
	No standard graphical representation of results leading to a lack of clear messaging and possibility of misinterpretation	A one-page report with graphical display of the results is created automatically and presented by the team at the conclusion of the audit
	Paper-based tool in English only	Multi-language e-Tool reduces language barriers to improve communication and accessibility
Data management	Data not available electronically for accessibility and data analysis	Data are available for extraction and statistical analysis
	Difficulty combining results from repeated audits	e-Tool combines and compares results across audits and displays data
	Difficult and time-consuming to combine results from multiple laboratories to create country-level graphics and summaries	e-Tool automatically combines data from multiple laboratories into one graphical representation, with summary statistics, for country-wide program management
	Scanning and storing paper copies of results	Electronic storage is convenient for multiple audits and sharing of information with stakeholders

SLIPTA, Stepwise Laboratory Quality Improvement Process Towards Accreditation.

**FIGURE 1:**
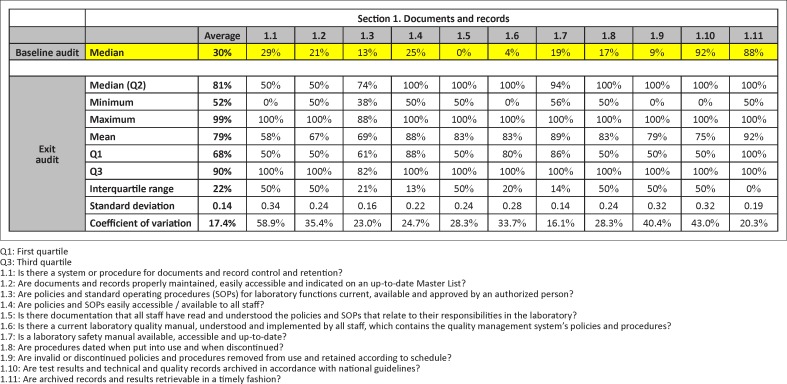
Example of e-Tool data output for baseline (row 1) and exit audit (other rows) for Section 1 (Documents and Records) compiled from 12 laboratories in Vietnam, 2013.

In addition to improved functionality of data at the laboratory-level, the e-Tool improves usability for country-level programme management. The ability to merge data easily from multiple laboratories affords an accurate analysis with robust statistical indicators, including means and medians, minimum and maximum values, interquartile ranges, standard deviations of means and coefficients of variation at both indicator and section levels. Compiling results electronically for multiple laboratories allows the remaining gaps to be addressed by appropriate improvement projects in the SLMTA programme and shared with MOH leadership (Figure 1).

**FIGURE 2:**
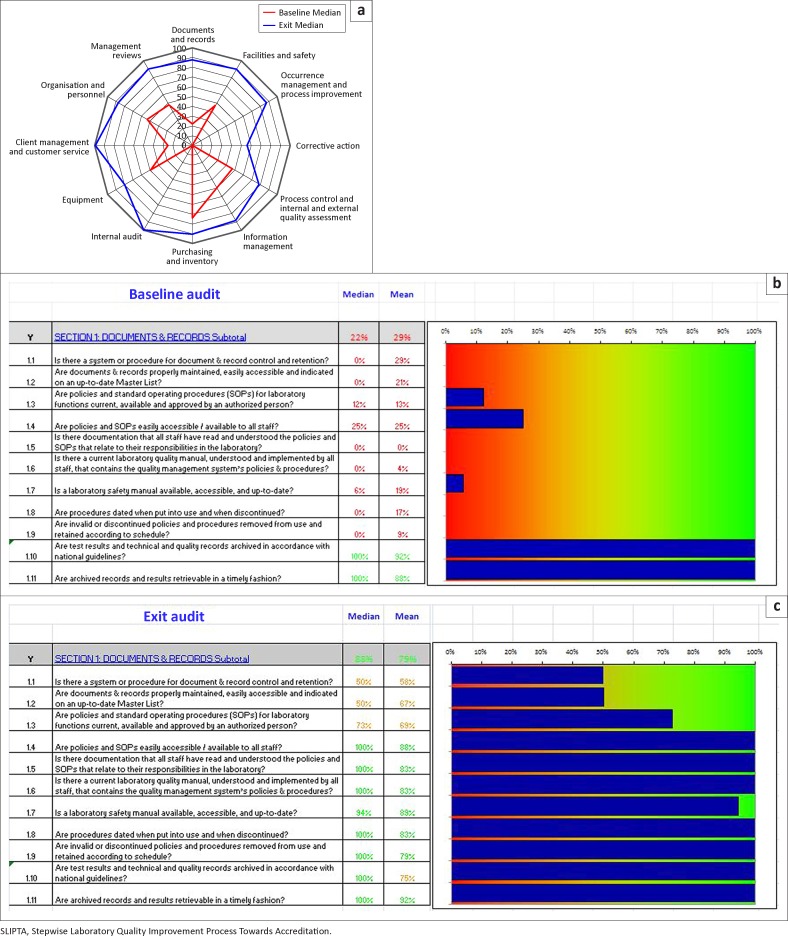
Visualised results for 12 laboratories in Vietnam using the SLIPTA e-Tool. (2a) Spider plot of combined baseline (May 2012) and exit (June to July 2013) audit median data for all 12 laboratory sites. (2b) Computer screen shot of baseline audit data for Section 1 (Documents and Records) of the SLIPTA checklist with bar-chart results. Colours indicate results that are acceptable (green), unacceptable (red) or partially compliant (yellow). (2c) Computer screen shot of exit audit data for Section 1 (Documents and Records) of the SLIPTA checklist with bar-chart results.

Data collected from all laboratories can be collated and used to assess countrywide gaps and initiate corrective actions. Figure 2 presents a sample e-Tool report, which combines results from all 12 participating laboratories in Vietnam. Baseline audits showed major gaps in four sections: internal audit (0%), corrective action (0%), occurrence management and process improvement (0%) and documents and records (22%) (Figure 2a). Section-level reports break out details of each question. For example, the Section 1 issues pertained predominantly to the development of manuals and Standard Operating Procedures (SOPs) (Figure 2b). Additional training was conducted on SOPs for the laboratory system, lectures on development of manuals and internal audit were included in the third SLMTA workshop and improvement projects related to these gaps were organised. Exit audit results showed substantial increases in the scores of these sections (100%, 57%, 87% and 88%, respectively) (Figure 2a), as well as in the subsection scores for Section 1 (Figure 2c). The clear visual summary of results provided by the e-Tool report facilitated timely development of an action plan to address the issues.

## Discussion

The newly-developed SLIPTA e-Tool improved the audit process, enhanced communication of audit results and ensured timely usability of the data collected in Vietnam and Cambodia.^7^ The e-Tool received positive feedback from in-country auditors for reducing workload. Because auditors are highly-trained limited resources, they must be utilised as efficiently and effectively as possible. Vietnam and Cambodia have only 10 to 12 qualified auditors in each country. As laboratory quality improvement scales up to include the more than 1700 public laboratories in Vietnam^8^ and 82 public laboratories in Cambodia,^9^ increasing the efficiency of the audit process will become even more critical.

The ability to leave a one-page printed summary in Vietnamese for each laboratory was a substantial improvement over the paper tool. The communication benefits of providing same-day, on-site language-appropriate results to laboratorians and administrators encourage engagement from leadership in the improvement process. Furthermore, presenting reliable results in a graphical format leads to better understanding of problems and use of data for improvement.

As with the TB-LAT e-Tool, this first version of the SLIPTA e-Tool has capacity for data exporting; however, some minor issues have been identified when transferring data between various versions of Microsoft^®^ Excel. Improvements to the tool should continue, based on feedback from end-users. In addition, studies are needed to provide a formal evaluation of the tool, including time and cost savings, improved accuracy and user preferences.

Potential benefits of the e-Tool expand beyond improved auditing. The tool incorporates auditing instructions for each SLIPTA question, which will enhance learning and consistency for laboratories performing internal audits. Furthermore, as additional auditors are trained, the e-Tool can be used for evaluation and validation of their competency and standardisation of audit criteria. Moreover, when unlocked, the electronic checklist can be customised and expanded to address additional laboratory- or country- specific data needs.

The SLIPTA checklist e-Tool is now available for use by other countries and can easily be customised as needed; for example, optional languages can be added for local settings. Widespread use of the tool will allow development of a database for SLMTA programme managers at the global level in order to evaluate systematic gaps in laboratory quality and to enhance overall planning, implementation and sharing of results.

## References

[1] World Health Organization’s Regional Office for Africa WHO guide for the stepwise laboratory improvement process towards accreditation in the African region [document on the Internet]. c2013 [cited 2014 Sep 20]. Available from: http://www.afro.who.int/en/clusters-a-programmes/hss/blood-safety-laboratories-a-health-technology/blt-highlights/3859-who-guide-for-the-stepwise-laboratory-improvement-process-towards-accreditation-in-the-african-region-with-checklist.html

[2] Yao K, Maruta T, Luman ET, et al The SLMTA programme: Transforming the laboratory landscape in developing countries. Afr J Lab Med. 2014;3(1), Art. #194, 8 pages. http://dx.doi.org/10.4102/ajlm.v3i1.194 10.4102/ajlm.v3i2.194 PMC4703335 26752335

[3] Yao K, Luman ET, SLMTA Collaborating Authors Evidence from 617 laboratories in 47 countries for SLMTA-driven improvement in quality management systems. Afr J Lab Med. 2014;3(2), Art. #262, 11 pages. http://dx.doi.org/10.4102/ajlm.v3i2.262 10.4102/ajlm.v3i2.262 PMC4706175 26753132

[4] Clinical and Laboratory Standards Institute Application of a quality management system model for laboratory services; Approved Guidelines – Third Edition. [CLSI document GP26-A3] Clinical and Laboratory Standards Institute; 2004.

[5] Integrated Quality Laboratory Services (IQLS) TB-LAT tuberculosis electronic laboratory assessment tool [document on the internet]. c2013 [cited 2014 Apr 28]. Available from: http://www.iqls.net/docs/TB-LAT_presentation.pdf

[6] Thuong NT, Pierson A, Hoang QTT, et al Vietnam SLMTA E-tool [document on the internet]. c2013 [cited 2014 Apr 28]. Available from: http://aslm.org/aslm2012/images/docs/Speaker_Presentations/Sunday_2_Dec/Symposium_Overview_Day1/2B_-_Vietnam_SLMTA_E-Tool.pdf

[7] Carvalho HM SLMTA in Vietnam – The roadmap for laboratory quality. Vietnam: US Centers for Disease Control and Prevention; 2012 (Unpublished).

[8] Ministry of Health of Vietnam Laboratory QMS in Vietnam – Roads towards improvement. Report presented at the Symposium on Quality Management for Medical Laboratories Bangkok, Thailand; 2011 March 17–18.

[9] Kingdom of Cambodia, Ministry of Health Appendix 17: Complementary package of activities for referral hospitals 2006–2010 In: National guidelines on complementary package of activities for referral hospital development from 2006 to 2010. 2nd ed., 2006; p. 128–130.

